# Omega-Class Glutathione Transferases Protect DNA from Oxidative Stress in Pathogenic Helminth Reproductive Cells

**DOI:** 10.3390/antiox12030560

**Published:** 2023-02-23

**Authors:** Jeong-Geun Kim, Insug Kang, Chun-Seob Ahn, Woon-Mok Sohn, Yoon Kong

**Affiliations:** 1 Department of Molecular Parasitology, Samsung Medical Center, Sungkyunkwan University School of Medicine, Suwon 16419, Republic of Korea; 2 Department of Biochemistry and Molecular Biology, Kyung Hee University School of Medicine, Seoul 02447, Republic of Korea; 3 Department of Parasitology and Tropical Medicine, Gyeongsang National University College of Medicine, Jinju 52727, Republic of Korea

**Keywords:** liver fluke, omega-class glutathione transferase, oxidative stress, redox signal, nuclear translocation, DNA, reproductive cells

## Abstract

Pathogenic helminths have evolved mechanisms to preserve reproductive function while surviving long-term in the host via robust protective responses. A protective role of antioxidant enzymes in preventing DNA degradation has long been proposed, but little evidence has been provided. Here, we show that omega-class glutathione transferases (GSTOs) are critical for maintaining viability by protecting the reproductive cell DNA of the carcinogenic liver fluke, *Clonorchis sinensis*. *Clonorchis sinensis* GSTO (CsGSTO) activities modified by changes in the GSH/GSSG and NADPH/NADP^+^ molar ratios suppressed the overproduction of reactive oxygen species. CsGSTO1 and CsGSTO2 catalyzed deglutathionylation under physiologic and low-stress conditions (GSH/GSSG ratio of 6:1 or higher) but promoted glutathionylation under high-stress conditions (GSH/GSSG ratio of 3:1 or lower). Gliotoxin-induced functional disruption of CsGSTOs in living *C. sinensis* reduced the GSH/GSSG molar ratio and increased the production of protein glutathionylation (PSSG) under physiologic and low-stress conditions, indicating that suppression of GSTO function did not affect deglutathionylation. However, the perturbation of CsGSTOs decreased the GSH/GSSG ratio but also reduced PSSG production under high oxidative stress, demonstrating that glutathionylation was impeded. In response to oxidative stimuli, *C. sinensis* decreased GSTO-specific dehydroascorbate reductase and thiol transferase activities and the GSH/GSSG ratio, while it increased the NADPH/NADP^+^ ratio and PSSG. CsGSTOs utilized GSH to regulate GSH/GSSG and NADPH/NADP^+^ recycling and triggered a redox signal leading to nuclear translocation. Nuclear-imported CsGSTOs were modified by glutathionylation to prevent DNA damage. Antibodies specific to CsGSTOs dose-dependently inhibited this process. Disruption of CsGSTOs or the depletion of GSH caused glutathionylation defects, leading to DNA degradation. Our results demonstrate that CsGSTOs and the GSH system play a previously unappreciated role in protecting DNA from oxidative stress.

## 1. Introduction

Glutathione transferases (GSTs) are involved in a variety of detoxification processes by catalyzing the conjugation of nonpolar toxic compounds containing electrophilic carbon, nitrogen, or sulfur atoms to reduced glutathione (GSH) [1]. These enzymes not only perform antioxidant functions that can form chain reactions against attack by endogenously and exogenously derived oxidants, but also contribute to cytoprotection via the transcriptional regulation of related genes, protein kinases, and signal pathways associated with stress responses [1,2,3].

Reactive oxygen species (ROS) disrupted DNA integrity and impeded pathways involved in DNA repair. Several different classes of GSTs have long been suggested to protect DNA from oxidative stress [4,5]. In response to oxidative stress, GST theta 1 (GSTT1) migrates to the nucleus and prevents DNA damage by suppressing the formation of DNA adductors generated by lipid peroxidation [6]. GST mu 1 (GSTM1), GST pi 1 (GSTP1), and GSTT1 vary in susceptibility to DNA repair pathways depending on genetic polymorphisms [7]. However, little information is available on how GSTs prevent DNA degradation or the redox signal mechanism mediating this phenomenon [4,5,6,7].

Omega-class GSTs (GSTOs), the most recently identified GST among several GST isoforms, exhibit distinct biochemical properties compared to other GSTs due to their unique topological characteristics [8]. GSTOs possess active site cysteine(s) capable of forming a disulfide bond with GSH, resulting in several atypical catalytic reactions, including dehydroascorbate reductase (DHAR), *S*-(phenacyl)glutathione reductase, and thioltransferase (TTase) activities [8]. It is postulated that GSTOs may be intermediate forms in the context of ancient glutaredoxin and the later GSTTs [9,10]. GSTOs play a multifaceted role essential for cell survival in a stressful milieu, which includes the activation of interleukin-1β, the regulation of signal molecules related to c-Jun-N-terminal-kinase (JNK)-mediated apoptosis, the modulation of mitogen activated protein kinase (MAPK), and the regulation of cell differentiation via MKK3/6 and p38 MAPK pathways [3,8,10,11,12].

*Clonorchis sinensis* is a small liver fluke that causes chronic infections in the hepatobiliary tract of humans and other mammals. Clonorchiasis is one of the most prevalent food-borne enzootic helminthiases in several Eurasian communities [13,14] and is a major risk factor for cholangiocarcinoma tumorigenesis in endemic areas [14,15]. The liver fluke is a group I biocarcinogen [16,17]. Histopathological examination of these patients frequently demonstrates characteristic features of intraductal papillary mucinous carcinoma [14,18]. Mechanical and chemical irritations by parasites, biliary ductal inflammations associated with ROS, perturbation and evasion of host cell apoptosis, and differentiation of host cholangiocytes are several of the mechanisms proposed in which they induce malignant transformation [19,20,21].

The liver fluke has well-adapted to humans and survives up to 30 years [22] in the hepatobiliary ductal lumen despite the presence of diverse environmental stressors that activate the redox signaling [23]. *Clonorchis sinensis* harbors a robust detoxifying system including 12 distinct GSTs in the genome [24]. The gene-specific induction of these GSTs exhibits an independent or coordinate expression profile suggesting a high specialization for detoxifying endogenous oxidants (sigma-GSTs; CsGSTSs) and conjugating xenobiotics in the extracellular environments (CsGSTMs and CsGSTSs). CsGSTOs show a target-specific adaptive response. CsGSTOs are specifically expressed in the reproductive system and play important roles in the protection of these organs during maturation and the response to oxidative stress [24,25,26]. *Clonorchis sinensis* appears to tightly regulate redox balance to respond to a variety of stressors, including oxidative stress.

We hypothesized that *C. sinensis* harbors antioxidant enzyme(s) capable of both glutathionylation and deglutathionylation, with a functional switch controlling vital cellular functions including cell viability. We previously showed that the knockdown of GSTO significantly reduced cell viability, whereas the introduction of GSTOs into these cells markedly, although not completely, restored cell survival upon oxidative stress [3]. These results led us to ask whether CsGSTOs are involved in DNA protection from oxidative stress. A protective role of GSTs in preventing DNA degradation has long been proposed, but little evidence has been provided [4,5,6,7]. This study shows that CsGSTO1 and CsGSTO2 catalyze deglutathionylation under physiologic and low oxidative stress conditions while they promote glutathionylation under high oxidative stress. CsGSTOs are translocated to the nucleus in response to oxidative stress, are modified by glutathionylation, and serve to prevent DNA degradation, thereby protecting *C. sinensis* reproductive cells that maintain parasitic fecundity.

## 2. Results

### 2.1. CsGSTOs Catalyze Both Glutathionylation and Deglutathionylation

We sought the antioxidant enzyme(s) responsible for both glutathionylation and deglutathionylation in *C. sinensis*. The glutathionylation of the recombinant form of CsGSTO1 (rCsGATO1), rCsGSTO2, rCsGSTM2, and rCsGSTS1 was tested using the deglutathionylated form of the SQLWCLSN peptide substrate [27] in the presence of 5 mM oxidized glutathione (GSSG). The tryptophan quenching assay revealed that the glutathionylation rate was significantly increased by all CsGSTs examined (Figure S1A). However, deglutathionylation was catalyzed only by rCsGSTO1 and rCsGSTO2, but not by rCsGSTM2 and rCsGSTS1 (Figure S1B). These results show that CsGSTOs uniquely catalyze both glutathionylation and deglutathionylation, whereas other CsGSTs only catalyze glutathionylation.

### 2.2. Cytosolic CsGSTOs Translocate to the Nucleus upon Oxidative Stimulation

CsGSTOs have been shown to play important roles in the protection of the reproductive organs such as egg, testes, vitelline follicles, and seminal receptacles with differential induction profiles [26]. We investigated the molecular biochemical changes that underlie this protective activity. Stimulation of the live worms with the oxidizing chemical, cumene hydroperoxide (CHP), did not significantly alter the total expression lev els of CsGSTO1 and 2 in eggs (CsGSTO1_contol_:CsGSTO1_stress_ = 1:0.96 and CsGSTO2_contol_:CsGSTO2_stress_ = 1:0.99), but resulted in a shift of the CsGSTOs from the primarily cytosol to cytosol and nucleus. 

**Figure 1:**
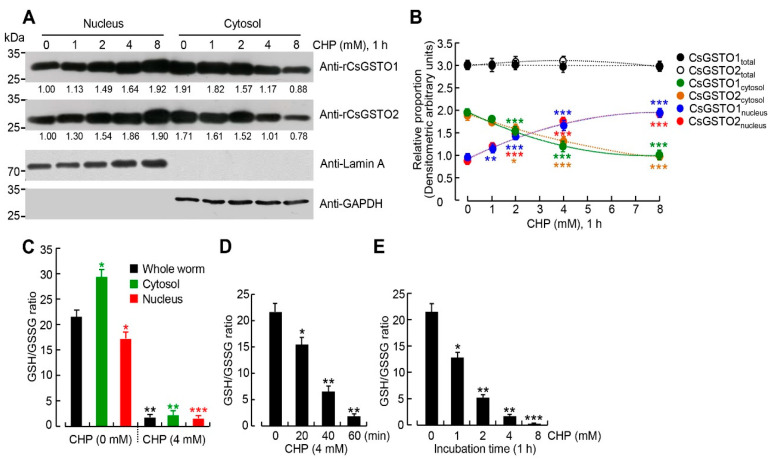
Oxidative stress induces nuclear translocation of cytosolic GSTOs in *Clonorchis sinensis* reproductive cells. (**A**) Dose-dependent nuclear translocation of cytosolic CsGSTO1 and 2 after treatment with varying doses of CHP (0–8 mM) for 1 h. Lamin A and GAPDH were used as nuclear and cytosolic markers. Numerals are mean values (*n* = 4) normalized to control (% of control nuclear extract) measured using ImageJ (https://imagej.nih.gov/ij/ accessed on 24 August 2022). (**B**) Changes in the relative proportions of CsGSTO1 and 2 in the nucleus and cytosol of worms treated with different CHP doses (0–8 mM) for 1 h. The intensity of Western blot bands, which are mean values of four independent experiments with three internal repeats, was quantified by ImageJ analysis (http://image.nih.gov/ij/ accessed on 24 August 2022). * *p* < 0.05, ** *p* < 0.01, *** *p* < 0.001 (Student *t*-test). (**C**) Changes in GSH/GSSG ratios of cytosolic and nuclear fractions upon treatment with 4 mM CHP for 1 h. Error bars represent SEM (*n* = 4). * *p* < 0.05; ** *p* < 0.01; *** *p* < 0.001 (Student *t*-test). (**D**,**E**) Shift of GSH/GSSG ratio in worms treated with 4 mM CHP for 0–1 h (**D**) and varying CHP doses from 0 to 8 mM for 1 h (**E**). Error bars represent SEM (*n* = 4). * *p* < 0.05; ** *p* < 0.01; *** *p* < 0.001 (Student *t*-test).

Treatment with varying doses of CHP for 1 h resulted in a dose-dependent nuclear translocation of cytosolic CsGSTOs. In the 8 mM CHP condition, the CsGSTO1 cytosolic/nuclear ratio shifted from 1:0.52 to 1:2.36 and that of CsGSTO2 from 1:0.58 to 1:2.55 (both *p* < 0.001) (Figure 1A,B). Furthermore, CHP induced a decrease in the GSH/GSSG ratio in the cytosolic and nuclear fractions (Figure 1C). Treatment with 4 mM or 8 mM CHP for 1 h reduced the GSH/GSSG ratio from 23:1 to 2:1 or from 23:1 to 1:1, which showed that redox potential of *C. sinensis* was remarkably altered in both the cytosol and nucleus with a dose-dependent manner (Figure 1D,E). It further suggests that changes in the GSH/GSSG ratio may cause the nuclear translocation of CsGSTOs. We investigated the redox signal mediating this shift and its pathophysiological relevance.

### 2.3. GSH and NADPH Redox Modify CsGSTO Function

**Figure 2:**
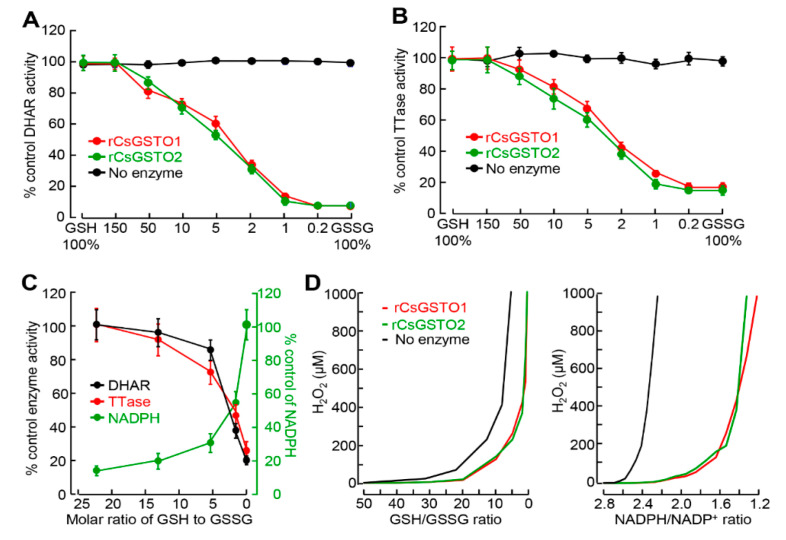
Glutathionylation modifies CsGSTO function. (**A**,**B**) Alteration of GSTO-specific DHAR (**A**) and TTase (**B**) activities by different GSH/GSSG molar ratios is shown using rCsGSTO1 and 2. Data are representative of four independent experiments. Error bars represent SEM. (**C**) Reciprocal changes of CsGSTO activities and NADPH levels by varying GSH/GSSG molar ratio. Data are representative of four independent experiments. Error bars represent SEM. (**D**) Changes in the GSH/GSSG (left panel) and NADPH/NADP^+^ (right panel) molar ratios were sensitive to peroxide concentration and modified CsGSTO activities to suppress H_2_O_2_ accumulation. Data are representative of three independent experiments.

GSH, a low-molecular-weight free thiol, is widely distributed in all subcellular compartments. GSH plays a crucial role in redox balance regulating key molecules and signal pathways associated with cellular protection under stressful conditions [11,28,29]. We speculated that changes in the GSH/GSSG molar ratio may affect the biological function of CsGSTOs by altering the redox state. We determined CsGSTO1 and 2 activities under different GSH/GSSG molar ratios using rCsGSTOs. GSH-dependent DHAR and TTase activities of rCsGSTOs were reduced at lower molar ratios (greater relative GSSG as under oxidative stress) by 82–89% at a 1:1 molar ratio of GSH/GSSG (Figure 2A,B). A low GSH/GSSG ratio induced a reciprocal increase in NADPH levels and decreased CsGSTO enzyme activities. At a GSH/GSSG ratio of 6:1 or higher, corresponding to physiologic and low stress under our experimental conditions, GSTO-specific DHAR and TTase activities were relatively stable, but decreased by 65–82% under the high stressful condition (GSH/GSSH ratio of 3:1 or lower) (Figure 2C). Conversely, supplementation with exogenous GSH dose- and time-dependently augmented rCsGSTO activities (Figure S2A–D). These results indicate that redox changes induce self-glutathionylation of CsGSTOs. As the surrounding environment is oxidized by GSSG, the thiol modification of both CsGSTOs increases, affecting CsGSTO enzyme activities possibly by altering tertiary/quaternary structures, as previously shown in GSTP [30].

We examined the contribution of CsGSTOs to the suppression of ROS production by measuring NADPH and H_2_O_2_ levels under various GSH/GSSG molar ratios. In the absence of rCsGSTOs, H_2_O_2_ accumulation was substantial (100 µM or more) even at GSH/GSSG and NADPH/NADP^+^ ratios of 20 and 2.6, whereas addition of rCsGSTO1 and 2 shifted these ratios to 12 and 2.8, respectively (Figure 2D). This result shows that CsGSTOs effectively suppress peroxide generation in the presence of sufficient GSH and NADPH.

### 2.4. The Nucleus Is Susceptible to Oxidative Stress and GSH Is Critical for Nuclear Transport of CsGSTOs

**Figure 3:**
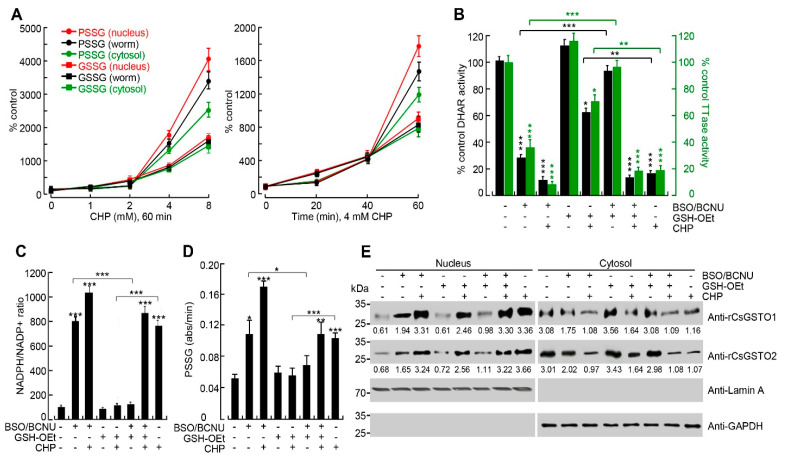
Nucleus is highly susceptible to oxidative damage and GSH is critical for nuclear translocation of CsGSTOs. (**A**) Oxidative stress induced increase in PSSG production. Live *C. sinensis* was exposed to different doses of CHP from 0 to 8 mM for 1 h (left panel). The worms were treated with 4 mM CHP from 0 to 60 min (right panel). All experiments were independently replicated in quadruplicate. Error bars represent SEM. (**B**–**D**) Changes in DHAR and TTase activities of CsGSTOs (**B**), NADPH/NADP^+^ levels (**C**), and PSSG levels (**D**) in *C. sinensis* depleted of GSH, with enhanced GSH, or with restored after depletion in the presence or absence of CHP (8 mM) for 1 h. Error bars represent SEM (*n* = 3). * *p* < 0.05; ** *p* < 0.01; *** *p* < 0.001 (Student *t*-test). (**E**) Immunoblot analysis of GSH-dependent nuclear translocation of cytosolic CsGSTOs. GSH-depleted, GSH-enhanced, or restored worms after depletion were incubated for 1 h in the presence or absence of CHP (8 mM). Numerals are relative expression levels measured by ImageJ (https://imagej.nih.gov/ij/ accessed on 24 August 2022). Lamin A and GAPDH were used for nuclear and cytosolic markers. All immunoblot images are representative of three independent experiments.

Oxidative damage increased both GSSG and protein glutathionylation (PSSG) in worms but the patterns were dissimilar each other. GSSG levels increased in the lag phase, with little effect on the severity and/or duration of the injury. Conversely, PSSG levels accelerated rapidly under high stressful condition in a dose- and time-dependent manner, and distribution pattern was rearranged. Treatment with 8 mM CHP for 1 h altered the GSSG proportions to a negligible extent in the whole worm, nucleus, and cytosol (100:102:98), but the PSSG proportions were changed to 100:120:82 in the respective compartments (relative expression levels of the control state was set at 100:100:100 in each compartment) (Figure 3A). Thus, the nuclear fraction of PSSG was substantially enhanced, indicating a greater decrease in the redox state in the nucleus than in the cytosol.

We studied the role of GSH in mediating this phenomenon. Live flukes treated with buthionine sulfoximine (BSO) plus 1,3-bis(2-chloroethyl)-1-nitrosourea (BCNU) to deplete GSH decreased CsGSTO-specific DHAR and TTase activities by approximately 90% and 70%, respectively, compared to untreated controls (all *p* < 0.001). However, restoration of GSH by GSH-reduced ethyl ester (GSH-OEt) following depletion increased DHAR and TTase activities by 3.2- and 3.7-fold, respectively, compared to the GSH-depleted or stressful condition (Figure 3B). The NADPH/NADP^+^ ratio was increased by the GSH depletion, but supplementation with excess GSH had little effect (Figure 3C). Depletion of GSH in worms caused an increase in PSSG production (*p* < 0.05) but returned to baseline levels upon restoration. Exposure of GSH-deficient worms to oxidative stress accelerated PSSG production but restoring GSH in these worms resulted in a partial, but significant, decrease in PSSG production (all *p* < 0.01, Figure 3D). Oxidative stress in GSH-deficient worms markedly increased nuclear transport of cytosolic CsGSTOs but restoring GSH did not inhibit this process (Figure 3E). The nucleus is more vulnerable to oxidative damage than the cytosol and responds sensitively to redox changes. Oxidation of GSH to GSSG by oxidative damage appears to trigger the nuclear translocation of CsGSTOs, responding to a severely altered redox state.

When we examined transcriptional changes of CsGSTOs in worms treated with BSO plus BCNU and GSH-OEt, no changes were observed. Their transcription levels were not altered with treatment of 8 mM CHP as well, albeit at uniformly lower GSH levels (Figure S3). These results confirm that post-translational thiol modification governs CsGSTO functions through the control of GSH and NADPH levels.

### 2.5. Gliotoxin Modulates CsGSTO Function

**Figure 4:**
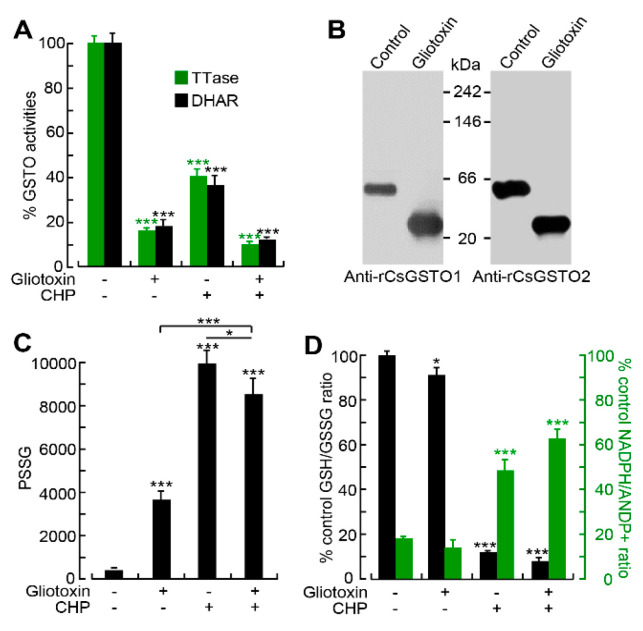
Gliotoxin-induced functional disruption of CsGSTO1 and 2 impairs glutathionylation. (**A**) Inhibition of CsGSTO activities by gliotoxin (100 μM) in the presence or absence of CHP (8 mM) for 1 h. Error bars represent SEM (*n* = 3). *** *p* < 0.001 (Student *t*-test). (**B**) Gliotoxin interfered with the formation of functionally active dimeric CsGSTOs. Figures are representative of three independent experiments. (**C**) Biphasic effects of gliotoxin on PSSG production in live *C. sinensis*. Flukes carrying normal GSTO activities and flukes whose GSTO function was impeded by gliotoxin (100 μM) were incubated for 1 h in the presence or absence of CHP (8 mM). Each value is the mean of three independent experiments with six internal repeats. Error bars represent SEM. * *p* < 0.05; *** *p* < 0.001 (Student *t*-test). (**D**) Alteration of GSH/GSSG and NADPH/NADP^+^ molar ratios by inhibition of CsGSTOs in live worms using gliotoxin (100 μM) in the presence or absence of CHP (8 mM) for 1 h. Each value is the mean of three independent experiments with six internal repeats. Error bars represent SEM. * *p* < 0.05; *** *p* < 0.001 (Student *t*-test).

We attempted to test the consequences of glutathionylation of CsGSTOs in live worms by inhibiting CsGSTOs, while GSTO inhibitor(s) acting on living organisms with sufficient specificity were not available. Since GSTO activity is glutathione-dependent, we used gliotoxin, a fungal-derived glutaredoxin-specific inhibitor [31]. Gliotoxin suppressed approximately 88% and 87% of DHAR and TTase activities of CsGSTO1 and CsGSTO2 by interfering with the formation of functionally active dimeric forms (Figure 4A,B). We confirmed that gliotoxin reduced CsGSTO enzyme activities in a dose- and time-dependent manner using rCsGSTOs (Figure S4A–D).

Gliotoxin decreased the GSH/GSSG molar ratio by 8.7% (*p* < 0.05), whereas it increased PSSG production by 15.9-fold (*p* < 0.001). Oxidative damage in worms in the presence of gliotoxin reduced the GSH/GSSG ratio similar to that in the absence of gliotoxin but resulted in a 14.3% decrease in PSSG production compared to worms exposed only to CHP (*p* < 0.05) (Figure 4C). Reciprocal increase in the NADPH/NADP+ ratio was observed (Figure 4D). These results indicate that the gliotoxin-induced suppression of CsGSTO activities interferes with glutathionylation and that CsGSTOs catalyze deglutathionylation under physiologic and low oxidative stress conditions while promoting glutathionylation under high oxidative stress.

### 2.6. Nuclear Accumulation of Glutathionylated CsGSTOs Prevents Oxidative DNA Damage

**Figure 5:**
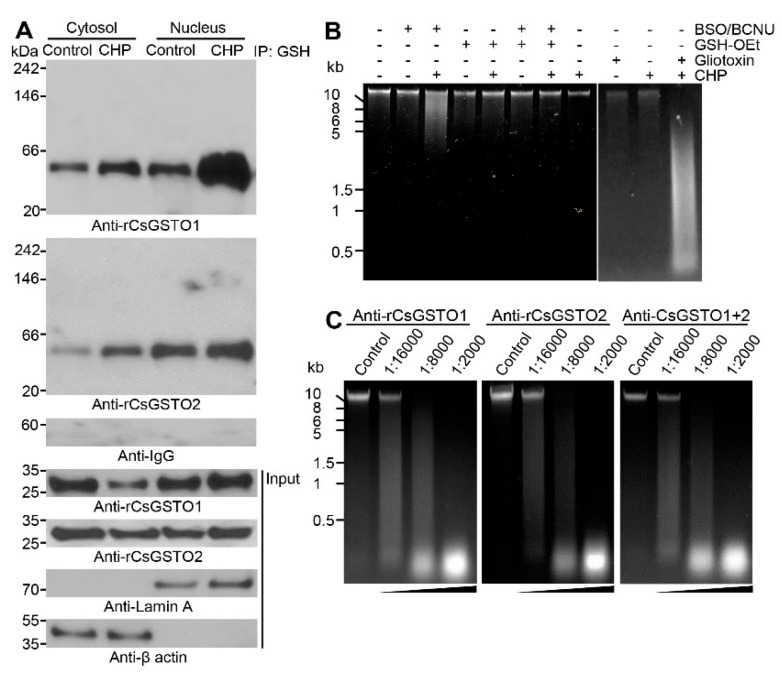
Glutathionylated CsGSTOs accumulated in the nucleus prevent oxidative DNA damage. (**A**) CsGSTO1 and 2 were immunoprecipitated with GSH, separated by 4–20% nonreducing SDS-PAGE, and probed with anti-rCsGSTO antibodies. Proteins for internal controls were separated by 12% SDS-PAGE under reducing conditions and detected by anti-rCsGSTO antibodies. Immunoblots probed with anti-Lamin A and anti-β-actin antibodies were used for nucleus and cytosol markers. Images are representative of three independent experiments. (**B**) Live *C. sinensis* treated with BSO + BCNU (200 μM each), GSH-OEt (2 mM), or gliotoxin (100 μM) were incubated for 1 h in the presence or absence of CHP (8 mM). Genomic DNAs extracted from each conditioned worm were analyzed by 1% agarose gel with ethidium bromide staining. Images are representative of three independent experiments. (**C**) Glutathionylated CsGSTO1 and 2 accumulated in the nucleus rescued DNA from oxidative stress-induced degradation. Genomic DNAs extracted from each conditioned worm as indicated were incubated with nuclear extract separately prepared from oxidatively damaged worms in the presence or absence of mono-specific antibodies against rCsGSTO1 and/or rCsGSTO2, after which separated by 1% agarose gel and stained with ethidium bromide. Images are representative of three independent experiments.

In response to oxidative stress, the expression levels of total CsGSTO1 and 2 were not significantly altered compared to controls (Figure 1A,B). However, the immunoexpression levels of glutathionylated forms were enhanced by 2.66-fold for CsGSTO1 and by 1.93-fold for CsGSTO2 relative to the respective control levels (both *p* < 0.01). The glutathionylated forms of CsGSTO1 and 2 were increased in both the cytosolic and nuclear fractions but were greater in the nucleus than in the cytosol, which demonstrates that glutathionylation of CsGSTOs occurs mainly after nuclear translocation (3.15- versus 2.18-fold for CsGSTO1 and 2.81- versus 2.44-fold for CsGSTO2, respectively) (all *p* < 0.01) (Figure 5A). In contrast, glutathionylation levels of other CsGSTs, i.e., CsGSTM2 and CsGSTS1, which also contributed to antioxidant protection [24,25], were not altered. These molecules were not transported into the nucleus (Figure S5). These collective results show that *C. sinensis* regulates glutathionylation in response to oxidative stress and this glutathionylation includes the glutathionylation of CsGSTOs in the nucleus.

Oxidative stress has been suggested as a major cause of DNA damage [4,5]. We asked whether glutathionylated CsGSTOs accumulated in the nucleus could protect DNA from oxidative injury. We stimulated live flukes with CHP under various conditions, including GSTO inhibition, GSH deficiency/excess, and GSH recovery after deprivation. We found that oxidative stress caused DNA degradation when GSH was deficient or when GSTO function was impaired (Figure 5B). We isolated genomic DNA from oxidatively damaged worms and co-incubated this DNA with nuclear fraction separately extracted from injured worms containing substantial amounts of glutathionylated CsGSTOs. We observed that DNA was not degraded but was well preserved. Conversely, block CsGSTO function by targeting antibodies specific to rCsGSTOs dose-dependently enhanced the breakdown of DNA (Figure 5C). These findings highlight that the *C. sinensis* genomic DNA is degraded when worms are subjected to oxidative stress in a state of insufficient nuclear CsGSTO function, either by glutathionylation defects or by the exhaustion of GSH.

## 3. Discussion

Helminths may utilize intrinsic mechanisms to maintain reproductive function. The protection of the reproductive organs from environmental stressors through inherent defensive factors is highly required. This study describes a physiological mechanism for the glutathionylation of the CsGSTOs and its role in protecting DNA from oxidative damage in the reproductive cells of *C. sinensis*, a pathogenic helminth associated with cholangiocarcinoma tumorigenesis [15,16,17]. The process by which CsGSTOs protect DNA from oxidative damage might occur in a two-step process, i.e., translocation into the nucleus and glutathionylation in the nucleus.

To decipher the role of CsGSTOs in protecting eggs from oxidative injury, we first analyzed the subcellular redistribution and biochemical changes of CsGSTOs before and after induced oxidative damage. When live flukes were subjected to oxidative stress, the worms sensitively responded to ROS and altered the GSH/GSSG and NADPH/NADP^+^ ratios (Figure 1). GSTO activities on GSH/GSSG and NADPH/NADP^+^ recycling are essential for regulating the redox balance in the changing micromilieu because the thiol group of cysteinyl residue supplied a reducing equivalent to generate GSSG in the reduced status and GSSG was reduced to GSH by NADPH in the oxidized state [32]. Excess GSH had little effect, indicating that limited amounts of GSH were oxidized to GSSG. The cellular redox environment might be dominated by enzyme-controlled process that promote the conversion of GSH rather than thermodynamic regulation of the GSH/GSSG couple (Figure 2C). We anticipate post-translational oxidative thiol modification is likely to occur in CsGSTOs.

In response to oxidative stress, both PSSG and GSSG were increased in worms but in different manners. Accumulation of PSSG was more pronounced than GSSG, especially when the worms were exposed to high stress or prolonged periods of time (*p* < 0.01). Depletion of GSH in worms also increased PSSG production. Moreover, PSSG levels were remarkably enhanced in the nuclear fraction (Figure 3A). This biochemical change might constitute the major driving force to induce nuclear translocation of cytosolic CsGSTOs. Nuclear CsGSTOs were modified by glutathionylation to counteract this unfavorable environment. Since a substantial amount of intracellular ROS generated by oxidative stress is scavenged by cytosolic antioxidants, the nucleus would be exposed to relatively low levels of ROS and may be more sensitive to ROS attack compared to other cellular organelles. ROS stress has actually been reported to be more cytotoxic to the nucleus than to other cellular compartments [33].

The contribution of glutathionylation to the biological function of CsGSTOs could be tested by pharmacological inhibition of enzymes. We analyzed the effect of gliotoxin, an inhibitor specific to glutaredoxin, since the TTase domain encompassed by the N-terminus of CsGSTOs exhibits glutaredoxin activity [26] and is principally associated with reduction of oxidative damage using GSH [34]. Glutaredoxin reacted with a glutathione thiyl radical to form a disulfide radical intermediate and transferred the radical to GSH to form GSSG or PSSG disulfide [35]. Nuclear transport of glutaredoxin 3 (Grx3) has been implicated in rescuing cells from oxidative stress-induced cell death [36]. The liver fluke, whose GSTOs were functionally disrupted by gliotoxin, showed a decreased GSH/GSSG ratio and increased PSSG production under physiologic and low stress conditions, indicating that suppression of the GSTO function did not affect deglutathionylation. However, when these worms were subjected to high oxidative stress, the GSH/GSSG ratio was decreased but the PSSG production was also decreased compared to those carrying normal GSTO function (Figure 4C,D), demonstrating that glutathionylation was inhibited. Glutathionylation mediated by cysteine sulfenylation plays a critical role in preventing irreversible oxidative damage caused by ROS [37]. The pathophysiological significance of glutathionylation in a wide range of antioxidant, anti-inflammatory, and antiapoptotic functions has been well established [34,38,39]. Glutathionylation of GSTP itself inhibited enzyme activity [30]. However, glutathionylation of GSTO and its consequences in DNA protection have not yet been addressed. Our results show that the glutathionylation of CsGSTOs reduces their catalytic activity but is essential for preventing oxidative stress-induced DNA damage.

In this study, oxidative stress, GSH depletion, and CsGSTO inhibition augmented PSSG production but did not cause DNA degradation. We speculate that DNA is well preserved, even though other organelles in the nucleus are susceptible to oxidative stress [32], as DNA conservation is intimately related to cell survival. DNA degradation occurs when *C. sinensis* suffers from oxidative stress under conditions of GSH depletion or inhibition of CsGSTO function. Blocking the inhibition of CsGSTOs or the supplementation with GSH protected against DNA damage (Figure 5B). When *C. sinensis*, having a normal GSTO function, is subjected to oxidative stress, CsGSTOs intervene and perform an appropriate role in DNA protection (Figure 5C). ROS can directly cause DNA damage, but this can be prevented by GSH [40]. Here, we describe the as-yet unknown and unique role of GSTO and provide prospects for new ways in which CsGSTOs utilize GSH to protect DNA from oxidative damage.

A previous study has reported that the ectopic expression of GSTO protects host cells. Transgenic *Caenorhabditis elegans* overexpressing the parasitic nematode *Onchocerca volvulus* GSTO-1 (OvGST3) demonstrated resistance to oxidative stress [41]. Rats infected with *C. sinensis* showed a time-dependent accumulation of CsGSTOs in host cholangiocytes [3]. CsGSTOs assimilated in host cholangiocytes exerts cellular protection via the regulation of several genes, protein kinases, and signaling pathways involved in antiapoptosis and cell proliferation, allowing cholangiocytes to maintain cell viability and exhibit adaptive responses [3]. This phenomenon might be related to clonorchiasis-associated cholangiocarcinoma tumorigenesis. Overexpression of GSTOs has been associated with breast and bladder cancer progression [42,43,44]. Another GST, GSTP1, increased autocrine growth on cancers with Kirsten rat sarcoma viral oncogene homologue (KRAS) and B-raf proto-oncogene serine/threonine kinase (BRAF) mutations [45]. The correlation between CsGSTO-mediated protection of host cholangiocyte DNA and the pathogenesis of cholangiocarcinoma should form the basis for future investigations.

Although this study shows that CsGSTOs are critically involved in protecting DNA from harsh stressful environments, our study includes several limitations. We expected that the restoration of GSH in GSH-depleted worms would significantly reduce the nuclear translocation of CsGSTOs upon oxidative stress, but we could not observe such effects (Figure 3E). Potentially, CsGSTOs could have already been imported to the nucleus during GSH depletion. This intriguing issue awaits further clarification. We could not ascertain a molecular mechanism for how CsGSTOs are imported to the nucleus. In response to oxidative stress, Grx3 is translocated into the nucleus via an as-yet unknown nuclear export signal in the C-terminal Grx3-G1 domain [36], whereas CsGSTOs did not possess such a domain [26]. Future studies are warranted to determine whether CsGSTOs utilize a receptor-mediated transport system or a novel nuclear import signal. Experiments employing a deletion mutant will also be needed to dissect this intriguing phenomenon. We currently do not know whether GSTO-mediated DNA protection is unique to *C. sinensis* or universal to other pathogenic helminths. In this regard, the small liver flukes, i.e., *Opisthorchis viverrini* and *O. felineus*, share many biological singularities with *C. sinensis*. These heterophyid trematodes show 85% sequence identity in the genome and 80.3% identity at the predicted protein-wide levels. They also have two GSTO isoforms [46,47]. The histopathological characteristics of the patients infected with these flukes are indistinguishably similar among them, showing inflammation-associated dysplasia, adenomatous hyperplasia and mucin-secreting metaplasia of biliary ductal epithelium, and ductal dilatation with periductal fibrosis [48,49]. Chronic infections with these flukes equally increased the risk of cholangiocarcinoma development in conjunction with oxidative injury [49,50,51]. Whether *O. viverrini* and *O. felineus* GSTOs are involved in DNA protection requires further elucidation.

## 4. Materials and Methods

### 4.1. Parasite

*Clonorchis sinensis* metacercariae collected from naturally infected freshwater fish were orally inoculated to 6-week-old Sprague-Dawley rats (Oriental Bio, Seongnam, Korea), regardless of sex, via a gavage needle at 100 metacercariae per rat. Animals were given free access to food and water. Adult worms were harvested from the bile ducts 2 months post-infection. Intact worms were individually collected under a dissecting microscope at 4 °C. To remove bile contaminants and cellular debris, the worms were washed at least 3 times each for 5 min with phosphate-buffered saline (PBS; 100 mM, pH 7.2) supplemented with a protease inhibitor cocktail (1 tablet/25 mL; catalog No. 1183617000, Sigma-Aldrich, St. Louis, MO, USA) at 4 °C. Animal experiments, including euthanasia of moribund animals, were performed in accordance with guidelines approved by the Ethical Committee in Samsung Medical Center, Sungkyunkwan University (2016-04-123) and Gyeongsang National University (GNU-16-015).

### 4.2. Chemical Treatment and Sample Preparation

Freshly isolated adult worms were stabilized for 1 h at 37 °C in serum- and phenol-red-free RPMI medium (catalog No. 11835030, ThermoFisher, Waltham, MA, USA) under 5% CO_2_ atmosphere. The groups of 10 worms in 1 mL fresh medium were treated with the indicated doses of cumene hydroperoxide (CHP; catalog No. 513296, Sigma-Aldrich; 1–8 mM for 1 h or 4 mM for 0–1 h), buthionine sulfoximine (BSO; catalog No. B2515, Sigma-Aldrich) plus 1,3-bis(2-chloroethyl)-1-nitrosourea (BCNU; catalog No. C0400, Sigma-Aldrich) (200 μM each per condition for 1 h), GSH-reduced ethyl ester (GSH-OEt; catalog No. G1404, Sigma-Aldrich; 2 mM for 1 h), and gliotoxin (catalog No. G9893, Sigma-Aldrich; 10–100 μM for 10 min–1 h) at 37 °C. Experimental worms were harvested and separated into nuclear and cytosolic fractions using the NE-PER Extraction Reagent Kit (catalog No. 78835, ThermoFisher) according to the manufacturer’s instructions. Proteins from each compartment were extracted in PBS (100 mM, pH 7.4) containing a protease inhibitor cocktail (1 tablet/25 mL). Nuclear and cytosolic fractions obtained from worms incubated without chemical treatment were used as controls.

### 4.3. Recombinant Proteins and Specific Antibodies

Recombinant proteins of CsGSTO1 (ANK78262), CsGSTO2 (ANK78263), CsGSTM2 (L47992), and CsGSTS1 (AF051318) were expressed in prokaryotes using gene-specific primers (Table S1) as described previously [24,25,26]. Bacterial endotoxins and lipids in recombinant proteins were removed using the Endotoxin Removal System (catalog No. L00350C, GenScript, Piscataway, NJ, USA) and Octyl-Sepharose 4B Fast Flow Column (catalog No. 68652-09-5, Sigma-Aldrich), respectively. Monospecific antibodies against each recombinant protein were generated using 6-week-old specific pathogen-free BALB/*c* mice [24,25,26]. IgG fractions were isolated using the Protein G-Sepharose 4B Fast Flow Column (catalog No. P3296, Sigma-Aldrich).

### 4.4. Tryptophan Quenching Assay

Substrate peptide (SQLWCLSN, Hanwol Tech, Seoul, Korea) was dissolved in ammonium acetate buffer (100 mM, pH 8.0) and glutathionylated in a 10-fold molar excess of oxidized glutathione (GSSG; catalog No. G4501, Sigma-Aldrich) for 24 h at 25 °C. Deglutathionylation of the substrate peptide was detected by observing the change in tryptophan fluorescence in McIlvaine buffer (200 mM Na_2_HPO_4_ and 100 mM citric acid, pH 7.0) supplemented with 0–4 mM reduced glutathione (GSH; catalog No. G6013, Sigma-Aldrich), 50 μM NADPH (catalog No. N5130, Sigma-Aldrich), 0 or 20 nM glutathione reductase (catalog No. G9297, Sigma-Aldrich), 1 mM EDTA (catalog No. 03609, Sigma-Aldrich), 0–20 μM substrate peptide, and 0–200 nM recombinant CsGSTs (rCsGSTs) [52]. Glutathione reductase and rCsGSTs were diluted in McIlvaine buffer containing 1 μg/mL bovine serum albumin (BSA). For glutathionylation assays, fluorescence measurements were performed in McIlvaine buffer supplemented with 5 mM GSSG, 1 μg/mL BSA, 1 mM EDTA, 5 μM substrate peptide, and 0–200 nM rCsGSTs [27]. All assays were independently performed in triplicate at 25 °C (excitation 280 nm; emission 356 nm).

### 4.5. GSTO Enzyme Assay

Formation of ascorbate by GST-dependent dehydroascorbate reductase (DHAR) activity was detected in potassium phosphate buffer (50 mM, pH 7.2) supplemented with 1 mM GSH and 0.25 mM dehydroascorbate (DHA; catalog No. 261556, Sigma-Aldrich) by measuring absorbance changes at 265 nm. Thiol transferase (TTase) activity was assayed by the absorbance changes at 340 nm using 2 mM hydroxyl ethyl disulfide (HEDS; catalog No. 38047, Sigma-Aldrich) in potassium phosphate buffer (50 mM, pH 7.2) containing 0.2 mM NADPH, 0.5 mM GSH, and 0.5 U glutathione reductase. One unit of enzyme activity was defined as the amount of enzyme that catalyzed the formation of 1 µM of product per min [26]. All enzyme assays were performed in triplicate for 2 min at 25 °C. Data were analyzed by best fit algorithm in SigmaPlot10.0.1 (Systat, Palo Alto, CA, USA).

### 4.6. NADPH/NADP^+^ Ratio Measurement

NADPH was measured after heating the samples for 30 min at 60 °C to decompose NAD^+^. To determine the NADPH/NADP^+^ ratio, worms collected on ice were lysed in extraction buffer by two cycles of freezing/thawing for 10 min, followed by thawing for 10 min at 37 °C. Then, lysate was mixed with the NADPH developer of a NADPH/NADP^+^ Assay Kit (catalog No. ab65349, Abcam, Cambridge, UK) and incubated for 4 h while monitoring absorbance at 450 nm on a microplate reader (Biotek, Santa Clara, CA, USA).

### 4.7. Determination of GSH and GSSG Levels, and GSH/GSSG Ratio

Protein lysates prepared by repeated freezing/thawing were precipitated with 5% sodium metaphosphoric acid (catalog No. 79613, Sigma-Aldrich). GSH levels in the supernatants were measured by incubating with *o*-phthalaldehyde (OPA; catalog No. P0657, Sigma-Aldrich) for 15 min. Absorbance was detected at 420 nm from 340 nm excitation. To determine GSSG levels, samples were incubated with *N*-ethylmaleimide (NEM; catalog No. 04259, Sigma-Aldrich) for 20 min and OPA for an additional 15 min before reading the fluorescence. The results for each sample are expressed as nmol/mg protein in the original extract.

### 4.8. PSSG Measurement

PSSG was measured by modifying the method previously described [27]. Briefly, protein pellets obtained by treating lysates with 5 g/L trichloroacetic acid (TCA; catalog No. T6399, Sigma-Aldrich) were washed 3 times with TCA (5 g/L) and 1 mg/mL Na_2_EDTA (catalog No. E5134, Sigma-Aldrich), incubated with 0.5 mmol/L dithiothreitol (DTT; catalog No. D0632, Sigma-Aldrich), and conjugated to fluorescent monobromobimane (catalog No. B4380, Sigma-Aldrich; 40 mmol/L dissolved in methanol). Samples were eluted isocratically with acetate buffer (0.25 mL/L, pH 3.09) containing methanol (200 mL/L) by high-pressure liquid chromatography (Agilent Technologies, Santa Clara, CA, USA). The fluorescence emission of derivatized thiols was measured at 480 nm from 390 nm excitation.

### 4.9. Quantitative Real-Time RT-PCR (qRT-PCR)

After the indicated treatments, experimental worms were harvested and washed with physiological saline at 4 °C and soaked immediately in QIAzol solution (catalog No. 79306, Qiagen, Hilden, Germany). Total RNA was extracted using the RNeasy Mini Kit (catalog No. 74104, Qiagen). Total RNA (200 ng) was treated with DNase and reverse-transcribed into cDNA using the iScript cDNA Synthesis Kit (catalog No. 12012801, Bio-Rad, Hercules, CA, USA), followed by the qRT-PCR analysis of *CsGST* expressions using gene-specific primers (Table S1) and the Rotor-Gene SYBR Green PCR Kit (catalog No. 204074, Qiagen) on a Rotor-Gene Q Real-time PCR System according to the manufacturer’s instructions. The qRT-PCR program included predenaturation for 5 min (94 °C), 40 cycles of amplification (94 °C for 15 s, 60 °C for 30 s, and 72 °C for 30 s), and a melt cycle from 65 °C to 95 °C. Expression was evaluated in three independent biological samples with 3 technical repeats and normalized to *C. sinensis* tubulin expression (ΔC_T_). Fold induction (ΔΔC_T_) was calculated by comparison to non-stimulated controls. Data were analyzed with the Rotor-Gene Q ScreenClust HRM software using the 2^−ΔΔC^_T_ method [53].

### 4.10. Western Blot

Detergent-dissolved crude, nuclear, and cytosolic extracts (100 μg per sample) were loaded onto a 12% polyacrylamide gels under denaturing conditions. After SDS-PAGE separation, the proteins were blotted onto nitrocellulose membranes (catalog No. sc-201706, Santa Cruz Biotechnology, Dallas, TX, USA) and probed with respective antibodies overnight at 4 °C. Hybridization bands were revealed with horseradish peroxidase (HRP)-conjugated host-specific IgG antibodies (1:1000–1:4000 dilutions) (catalog Nos. 0855550 (anti-mouse IgG) and 08674371 (anti-rabbit IgG), MP Biomedicals, Santa Ana, CA, USA) and quantified using the West-Q Pico Enhanced Chemiluminescence Kit (catalog No. W3652-050, GenDEPOT, Sigma-Aldrich). Expression of *C. sinensis* tropomyosin (CsTrop) was measured as the gel loading control. Detection of glyceraldehyde-3-phosphate dehydrogenase (GAPDH), β-actin, and Lamin A proteins was used for cytosolic and nuclear markers. Band intensities were determined using ImageJ (https://imagej.nih.gov/ij/ accessed on 24 August 2022).

Antibodies used and dilution factors were as follows: Anti-rCsGSTO1 and 2 (1:1000 dilution), anti-rCsTrop (1:100 dilution), anti-rCsGSTM2 (1:400 dilution), anti-rCsGSTS1 (1:1000 dilution), anti-glutathione (clone D8, catalog No. 101-A, ViroGen, Boston, MA, USA; 1:200–1:1000 dilutions), monoclonal anti-Lamin A (ab108595, abcam; 1:200 dilution), monoclonal anti-GAPDH (clone 1D4, catalog No. MA1-16757, ThermoFisher; 1:200 dilution), and anti-β-actin (clone AC-15, catalog No. AM4302, ThermoFisher; 1:400 dilution). All images were obtained after a 2 min exposure for quantitative analysis.

### 4.11. Immunoprecipitation

Worm extract (100 μg per sample) was immunoprecipitated with anti-glutathione antibodies (5 μL per sample, ViroGen), or IgG antibody (10 μL per sample), and subsequently with 50 μL rProtein A + G agarose 4B (catalog No. 20423, Pierce) overnight at 4 °C. Bound proteins were eluted with glycine buffer (50 mM, pH 3.0). The pH of the eluent was immediately adjusted to pH 7.2 by adding 1 M Tris (catalog No. 10708976001, Sigma-Aldrich). Proteins were separated by 12% reducing SDS-PAGE or 4–12% gradient nonreducing SDS-PAGE, then probed with monospecific antibodies against rCsGSTO1 (1:1000 dilution), rCsGSTO2 (1:1000 dilution), rCsGSTM2 (1:400 dilution), or rCsGSTS1 (1:1000 dilution), respectively. Immunoreactive bands were detected with the West-Q Pico Enhanced Chemiluminescence Kit (Sigma-Aldrich) as described above.

### 4.12. Amplex Ultra Red Assay

A reaction mixture (100 μL) which contained 50 μM Amplex Red reagent (catalog No. A22177, Invitrogen, Waltham, MA, USA) in Krebs-Ringer phosphate buffer (145 mM NaCl, 5.7 mM Na_2_HPO_4_, 4.86 mM KCl, 0.54 mM CaCl_2_, 1.22 mM MgSO_4_, 5.5 mM glucose, and 0.1 U/mL HRP, pH 7.35) and varying molar ratios of GSH:GSSG from 50:1 to 1:1 were treated with rCsGSTO1 or 2 (50 μg per reaction), after which the samples were shielded from light and incubated for 30 min at 25 °C. Then, fluorescence emission at 590 nm from 565 nm excitation was measured using a microplate reader (Biotek).

### 4.13. DNA Protection Assay

Adult *C. sinensis* worms exposed to various stressful conditions, including GSH deficiency by BSO + BCNU treatment (200 μM per sample), excess by GSH-OEt supplementation (2 mM), and inhibition of GSTO functions by gliotoxin treatment (100 μM), were incubated in the presence or absence of CHP (8 mM) for 1 h at 37 °C in a 5% CO_2_ incubator. DNAs from each conditioned worm were extracted using the DNeasy Blood and Tissue Kit (catalog No. 69504, Qiagen), eluted in 50 μL elution buffer, and quantified at 260 nm in a NanoDrop spectrophotometer (ThermoFisher). Degradation of respective DNAs was examined by 1% agarose gel electrophoresis with ethidium bromide staining.

To evaluate the protective role of CsGSTOs against oxidative-stress-induced DNA damage, we extracted nuclear fraction of live worms treated with 8 mM CHP for 1 h at 37 °C using NE-PER Extraction Reagent Kit (ThermoFisher). The nuclear fraction (200 μg per sample) was incubated with different dilutions (from 1:2000 to 1:16,000 in PBS) of antibodies specific to rCsGSTO1 and/or 2 for 5 min at 37 °C. The reaction mixture was then coincubated with genomic DNA (50 μg per sample) separately isolated from oxidatively injured worms for 5 min at 37 °C. DNA degradation under each condition was analyzed by 1% agarose gel electrophoresis and ethidium bromide staining.

### 4.14. Statistical Analysis

All experiments were performed 5 times with at least 2 technical repeats per samples. Data are presented as the mean ± standard error of the mean (SEM). Treatment group means were compared by one-way analysis of variance (ANOVA) with Student *t*-tests for pair-wise comparisons. Differences were considered to indicate significance at * *p* < 0.05, ** *p* < 0.01, and *** *p* < 0.001 for all tests.

## 5. Conclusions

Helminth exploits an intrinsic mechanism to preserve reproductive functions in stressful host environments. Antioxidants have long been theoretically proposed to protect DNA from oxidative damage, but little evidence has been provided [4,5]. CsGSTOs are sensitive to the generated ROS and inhibit ROS accumulation by changing the GSH/GSSG and NADPH/NADP^+^ molar ratios. CsGSTOs catalyze deglutathionylation under physiologic and low-stress conditions but promote glutathionylation under high-stress conditions. Disruption of CsGSTOs does not affect deglutathionylation under physiologic and low-stress conditions, while impeded glutathionylation at high-stress conditions. CsGSTOs utilize GSH to regulate GSH/GSSG and NADPH/NADP^+^ recycling and translocate to the nucleus. Nuclear CsGSTOs are modified by glutathionylation to prevent oxidative DNA damage. Disruption of CsGSTOs or depletion of GSH causes glutathionylation defects, resulting in DNA degradation.

**Figure 6:**
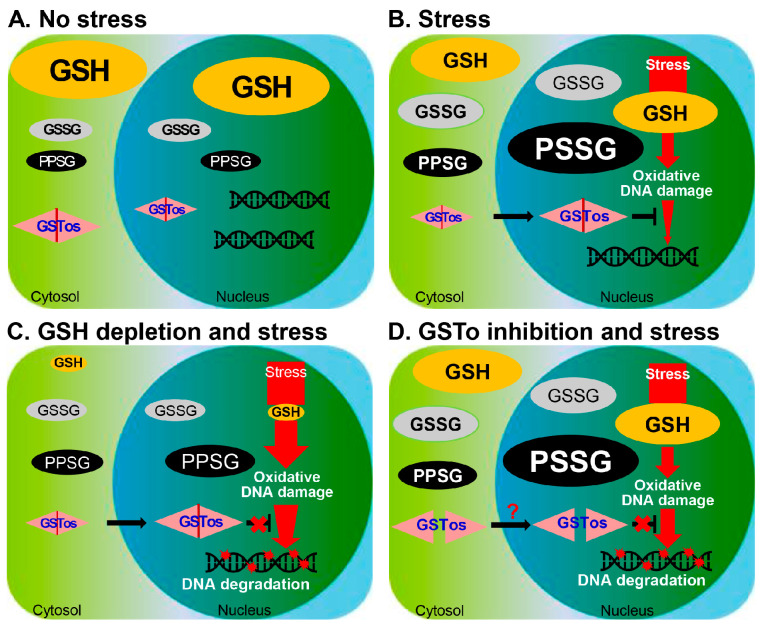
Schematic representation of CsGSTO-mediated DNA protection from oxidative damage. (**A**) In the steady state, a large portion of CsGSTOs is present in the cytosol and a small portion in the nucleus. GSH, GSSG, and PSSG are distributed in the cytosolic and nuclear compartments with homeostatic balance. (**B**) When liver flukes suffer from oxidative stress, redox potentials decrease in both the cytosol and nucleus, with a greater extent in the nuclear compartment. Alterations in GSH, GSSG, and PSSG levels are accompanied. Significant amounts of CsGSTOs translocate to the nucleus to facilitate homeostatic adaptation and cytoprotection. Nuclear-accumulated CsGSTOs are modified by glutathionylation to protect against DNA damage. (**C**) When worms deficient in GSH by certain predisposing factor(s) are exposed to oxidative stress, PSSG production is not prominent due to impaired GSH/GSSG recycling. Cytosolic GSTOs translocate to the nucleus, but nuclear CsGSTOs cannot prevent DNA degradation due to the glutathionylation defects because of insufficient GSH. (**D**) When GSTOs are functionally disrupted by dimerization defects, DNA degradation occurs upon oxidative damage.

Based on our collective findings, we propose a mechanism for GSH-mediated DNA protection by CsGSTOs from oxidative stress (Figure 6A–D). When liver fluke experiences oxidative stress, the redox potential is decreased in both the cytosol and nucleus by alterations in GSH, GSSG and PSSG levels, and to a greater extent in the nucleus. Significant amounts of GSTOs translocate to the nucleus to facilitate homeostatic adaptation. Nuclear CsGSTOs are modified by glutathionylation and protect DNA damage. When GSH is exhausted in worms by certain predisposing factors, PSSG production is substantially increased in the cytosol and nucleus, especially in the nucleus, owed to the impairment of GSH/GSSG recycling. Cytosolic CsGSTOs translocate to the nucleus, but nuclear CsGSTOs cannot prevent DNA degradation due to insufficient GSH. When GSTOs are functionally disrupted by dimerization defects, DNA degradation occurs upon oxidative stress. Our results show that the nuclear translocation of CsGSTOs followed by glutathionylation is essential for preventing oxidative DNA damage and offers the prospect of new ways to protect against such stress.

## Data Availability

The data presented in this study are available within the article and its Supplementary Materials. Other data that support the findings of this study are available upon request from the corresponding author.

## Supplementary Materials

The following supporting information can be downloaded at: https://www.mdpi.com/article/10.3390/antiox12030560/s1. **Figure S1**. CsGSTO1 and 2 catalyze both glutathionylation and deglutathionylation. **Figure S2.** Changes in rCsGSTO activities by varying GSH/GSSG molar ratio. **Figure S3.** Transcriptional changes of CsGSTO1, CsGSTO2, CsGSTM2, and CsGSTS1 in the presence or absence of oxidative stress. **Figure S4.** Gliotoxin inhibits rCsGSTO activities in a dose- and time-dependent manner. **Figure S5.** Nuclear import of CsGSTM2 and CsGSTS1 following oxidative stress. **Supplementary Table S1.** Primers used for expression of recombinant proteins and qRT-PCR.
