# Supplementary material for: Omega-Class Glutathione Transferases Protect DNA from Oxidative Stress in Pathogenic Helminth Reproductive Cells
Source: Antioxidants (Basel). 2023 Feb 23;12(3):560. doi: 10.3390/antiox12030560 (PMC10045047; doi:10.3390/antiox12030560)
Supplement: Supplementary file 1 [file antioxidants-12-00560-s001.zip › antioxidants-2146252-supplementary.pdf]

Supplementary Table S1. Primers used for expression of recombinant proteins and qRT-PCR

Experiments	Gene name	Sequence (5'→3')
Expression of recombinant proteins	CsGSTO1-forward	ATGCCAACCTGTTCCAAGCATTTGCGACAAGGAGA
	CsGSTO1-reverse	TTACATGTCCCAGTCAGGATGACCACTTCGTATGG
	CsGSTO2-forward	ATGTGCTATCTGGGAGACGCAGGGACAGTGTCACG
	CsGSTO2-reverse	CTAGGCAATTTCAAGATTTGGCTTTCCAGCTTTCC
	CsGSTS1-forward	GGGGTCGACAGAGGATGATATTGCATGCAAA
	CsGSTS1-reverse	TAAGCGGCCGCTTACAGGGGCGTTTCTGGTC
	CsGSTM2-forward	AAAGTCGACAGCTGGAGTACGTCGGTGACAG
	CsGSTM2-reverse	TAAGCGGCCGCTTATTTCGGAGGAGCATCGC
qRT-PCR	CsGSTO1-forward	GTTTCCATTTGTGGAC
	CsGSTO1-reverse	TGGTAGCTGCAATACG
	CsGSTO2-forward	TCGTTTGAGCGAATCG
	CsGSTO2-reverse	CAGCGAGACTGAGTTG
	CsGSTS1-forward	TGATATTGCATGCAAACGGTG
	CsGSTS1-reverse	GAATCACCCATCATATGGAAG
	CsGSTM2-forward	GTTTGTTGCTGGAGTACGTC
	CsGSTM2-reverse	AGCAATGTAACGTAGAATGGC
	Cs tubulin-forward	ATTCAGCTGTCCTGGGAAAC
	Cs tubulin-reverse	ACTGCATTGATAACGAAGCG

Legends for supplementary figures

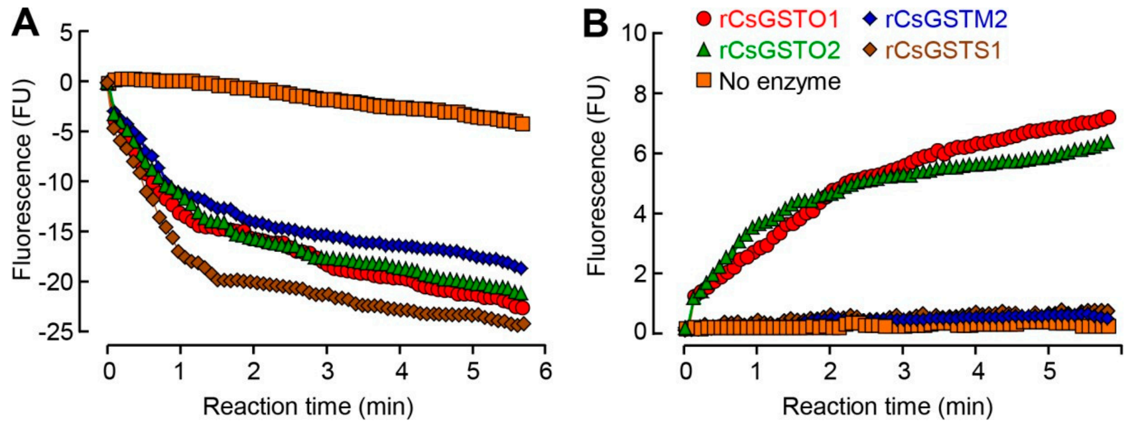

Supplementary Figure S1. CsGSTO1 and 2 catalyze both glutathionylation and deglutathionylation. rCsGSTO1 and rCsGSTO2 show both glutathionylation (**A**) and deglutathionylation (**B**), while rCsGSTM2 and rCsGSTS1 catalyze only glutathionylation against peptide substrates. Glutathionylation and deglutathionylation were monitored by tryptophan quenching assay. Figures are representatives for three independent assays.

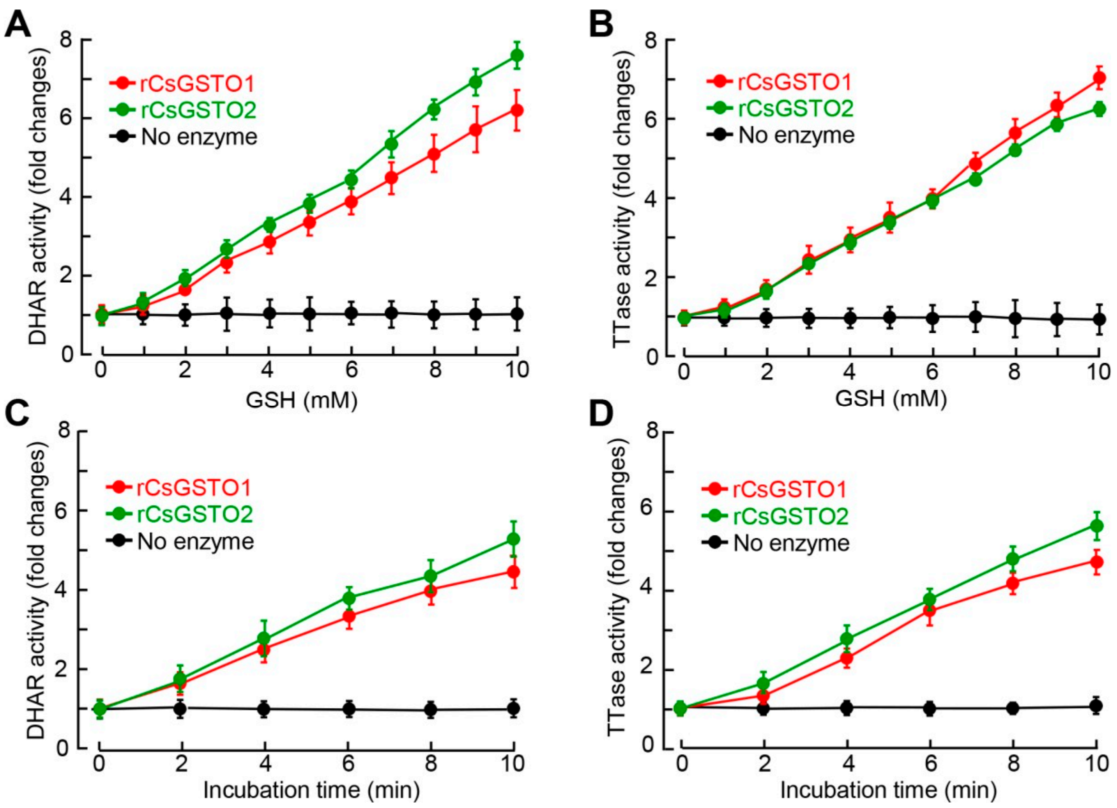

Supplementary Figure S2. Changes in rCsGSTO activities by varying GSH/GSSG molar ratio. Treatment with varying doses of GSH (0–10 mM) revised DHAR (**A**) and TTase activities (**B**) of rCsGSTO1 and 2. Time-dependent augmentation of DHAR (**C**) and TTase activities (**D**) of rCsGSTO1 and 2 by fixed concentration of GSH (4 mM). Error bars represent SEM (n = 4).

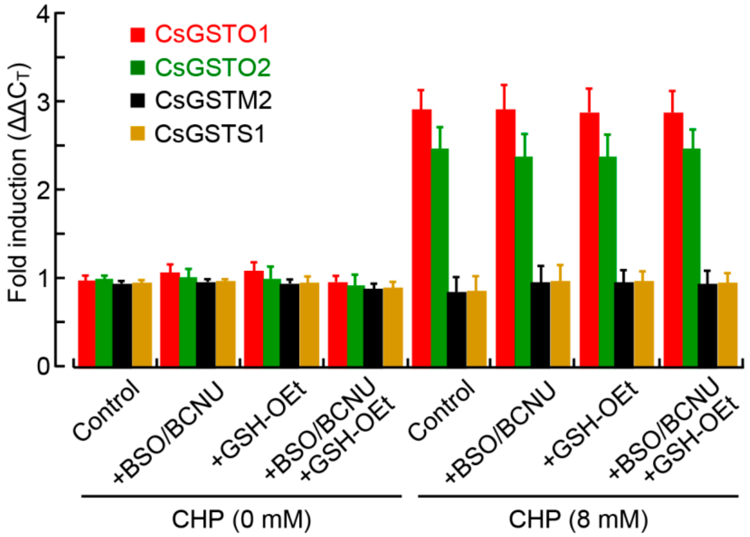

Supplementary Figure S3. Transcriptional changes of CsGSTO1, CsGSTO2, CsGSTM2, and CsGSTS1 in the presence or absence of oxidative stress. qRT-PCR shows that treatment with 8 mM CHP for 1 h in live flukes deficient in GSH by BSO + BCNU treatment (200 µM each), replenishment of excess GSH by GSH-OEt (2 mM), and restoring GSH after depletion did not affect the transcription levels of CsGSTs. Error bars represent SEM (n = 3).

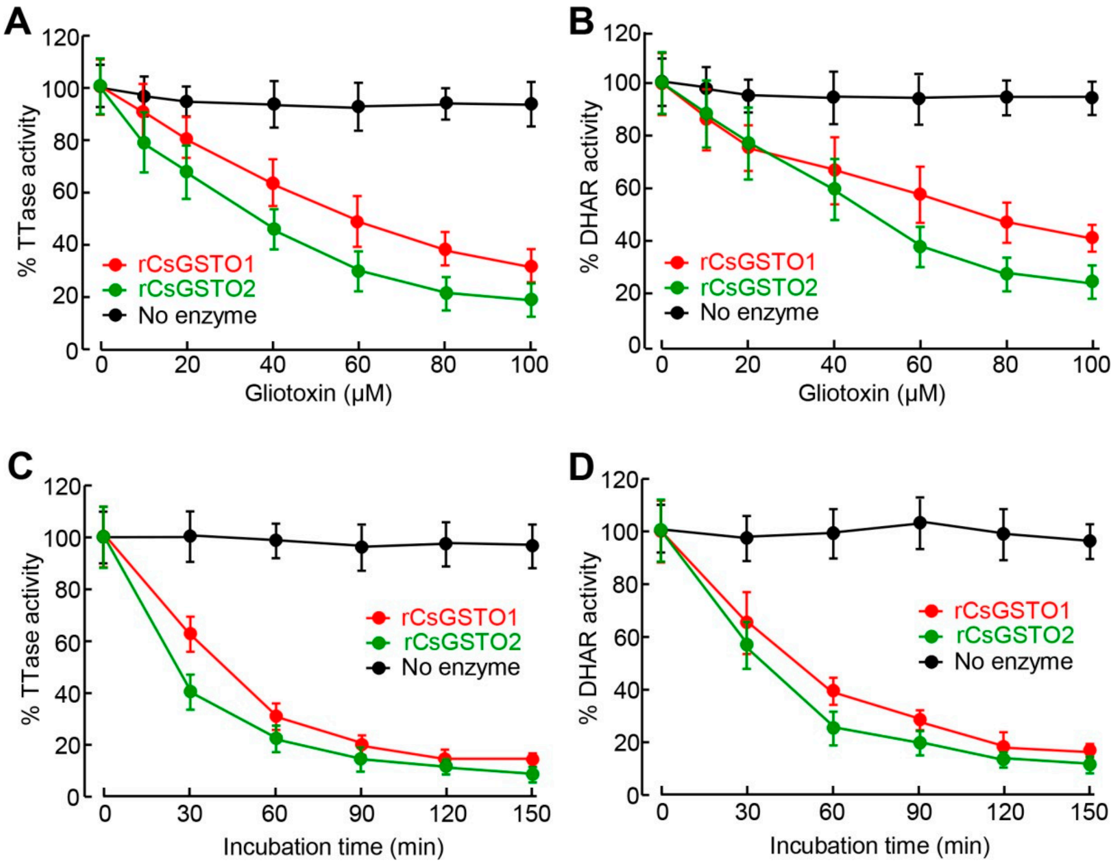

Supplementary Figure S4. Gliotoxin inhibits rCsGSTO activities in a dose- and time-dependent manner. (**A** and **B**) Changes in GSH-dependent TTase (**A**) and DHAR activities (**B**) upon treatment with different doses of gliotoxin (0–100 μM) for 1 h. Each value is the mean of three independent experiments with six internal replicates. Error bars represent SEM. (**C** and **D**) Time-dependent changes of TTase (**C**) and DHAR activities (**D**) by 100 μM gliotoxin. Each value is the mean of three independent experiments with six internal repeats. Error bars represent SEM.

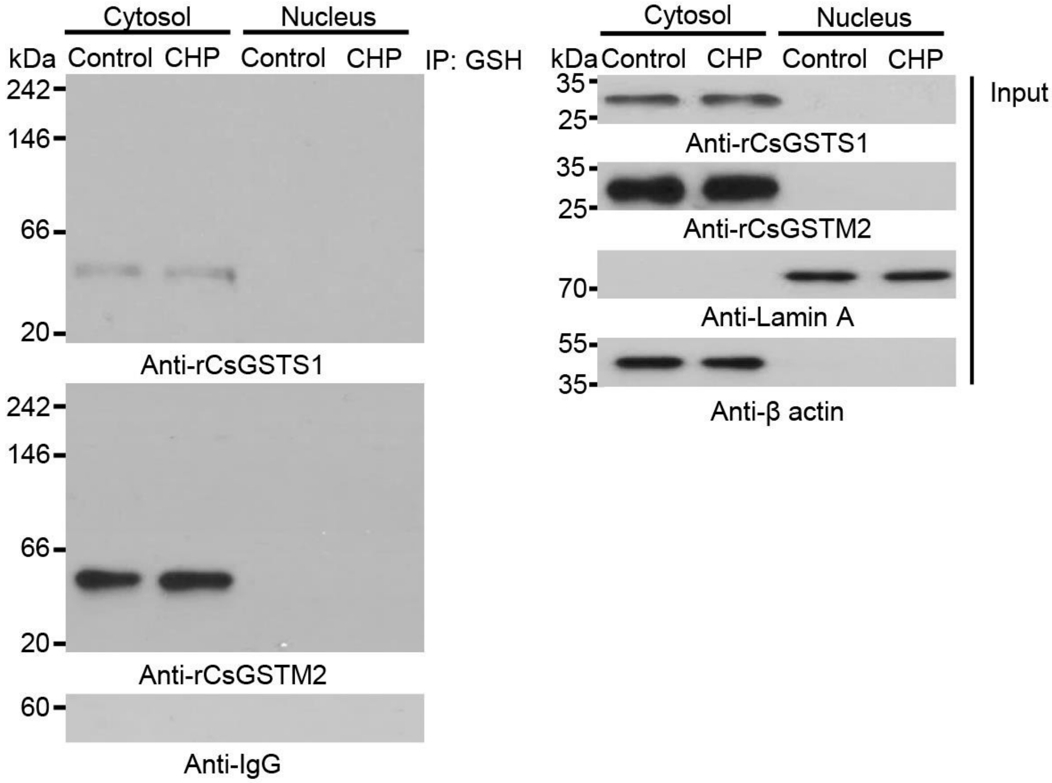

Supplementary Figure S5. Nuclear import of CsGSTM2 and CsGSTS1 following oxidative stress. CsGSTM2 and CsGSTS1 were immunologically precipitated with GSH, separated by 4%–20% non-reducing SDS-PAGE and proved with anti-rCsGSTM2 and anti-rCsGSTS1 antibodies. Proteins for nuclear (Lamin A) and cytosolic (β-actin) markers were separated by 12% reducing SDS-PAGE and probed with antibodies specific to respective proteins. Images are representative of three independent experiments.
